# Rat Kidney Cancers Determined by Dietary Ochratoxin A in the First Year of Life

**DOI:** 10.15586/jkcvhl.2016.58

**Published:** 2016-09-26

**Authors:** Peter Mantle

**Affiliations:** Centre for Environmental Policy, Imperial College London, London SW7 2AZ, UK

**Keywords:** Balkan endemic nephropathy, karyomegaly, latency, leukaemia, renal cell carcinoma, urothelial cancer

## Abstract

An experiment to explore renal carcinogenic efficacy of male rat exposure to dietary ochratoxin A (OTA) only in the first year of life has been made in comparison to lifetime exposure. Ten months exposure to OTA at 300 µg/kg b.w. was sufficient to cause high incidence of tumours which became apparent clinically after a latency of up to a year. As a putative model for human kidney cancer, the study shows a silent organ-specific carcinogenic effect through protracted exposure up to middle age and focused probably on very few nephrons. So far, tumourigenesis has not been recognised until in the last quarter of natural rat life, but for OTA, rat renal carcinogenesis requires both long exposure and only during the first year of normal longevity. The present findings offer an experimental framework within which systematic histopathology during tumourigenesis might show whether findings of mechanistic studies in key focal neoplasms can reasonably be applied to OTA as a putative renal carcinogen for idiopathic kidney cancer in humans. Already, the rat tumours mimic those occurring spontaneously in the Eker rat, and there is disparity between the large necessary OTA exposure in the rat and the trace amounts of OTA consumed by humans. In all such complex considerations it is important to adhere rigorously to established principles of disease epidemiology.

## Introduction

Ochratoxin A (OTA) is the most potent naturally-occurring nephrotoxin causing experimental kidney cancer in rats (1), and has been recognised as presenting the gold standard for log dose/response relationships as a thresholded carcinogen (2). The comprehensive design of the National Toxicology Program (NTP) study established that, whereas no renal cancer was evident after 9 months of the gavage dosing regimen, 15 months of gavage 5 days/week caused two cases of renal cancer amongst 15 males at the high dose (mean daily intake 150 µg/kg b.w.). In a larger lifetime study the OTA regimen for up to 2 years gave a 60 % incidence of renal cancer. Consequently, the NTP study has formed the principal experimental model for consideration of OTA as a cause of human kidney cancer.

In the context of a major European Union-funded programme (2001–2004) on OTA’s mode of carcinogenicity, opportunity arose to develop improved protocols for tumour expression and analysis. It was also intended to refine understanding of experimental optima for tumourigenesis to provide a better rat model for debate about human relevance. The first step was a male rat lifetime study in London on responses to continuous dietary administration of OTA (3), which revealed only a 20 % incidence of kidney cancer at a daily OTA intake similar to that at the high dose of the NTP study, if expressed as the average daily intake through a week (3). This expression is relevant to a commonly used unit in toxicity risk analysis, namely the tolerable weekly intake (TWI). The London study, and all subsequent ones there, used delivery nearer to natural contamination by including both the nephrotoxin and the mould that produced it, and voluntary dietary consumption enabled natural intestinal absorption over a longer period in a day than that consequent on oral gavage. Continuous dietary delivery also avoided the temporary decline in circulating OTA concentration in blood during the two weekly ‘rest days’ in applying the NTP’s 5-day gavage regimen (4) that was also reflected in ochratoxin B (OTB) excretion (5).

A further experiment in London provided an intake matching the NTP mid-dose (1), and has already been described (6). The present report focuses on the more striking pathological findings at what was, in effect, twice the intake of the NTP study high dose and which were made on animals that were exactly contemporary with those in the study by Mantle and Kulinskaya (6). The present report also represents the highest lifetime dietary OTA intake studied to date in F344 rats, and formed a context for exploring also the lifetime outcomes of the same dietary OTA exposure for only 10 months.

## Materials and methods

### Production of OTA and formulation for dietary experiment

Experimental design was generally the same as used previously (3, 6), with the second of which the present study was concurrent (6), so that the OTA source was identical. The UK source and experimental environment was the same for all animals. Thirty F344 male rats were used for the present study. Briefly, standardised OTA production was performed with *Aspergillus ochraceus* (7) on moist shredded wheat (40 g) in rotary shaken solid substrate fermentation in 500 ml conical flasks (Supplementary **Figure**) at 28 ᵒC for 2 weeks to yield batches of a product containing 5–6 mg OTA/g (5). Quality assessment of the product showed that in addition to OTA there was 5–10 % of OTB (des-chloro OTA) which is insignificantly bioactive as a mycotoxin; no other mycotoxins were produced in this fermentation. Each week, fermentation product was homogenised into powdered rat feed to dilute by the required factor of ~1000 to achieve the experimental OTA contamination of rat diet at 5 µg/kg. Young rats were caged in groups of five and daily given 100 g feed in an aluminium foil container, providing 20 g/rat at 300 µg OTA/kg b.w. Consequently, condition of all animals could be inspected daily. Caged groups were reduced during subsequent growth to a maximum of three. All handling and procedures were carried out in accordance with the UK Animals (Scientific Procedures) Act 1986. Occasional blood samples (200 µl) were taken from a tail vein under mild general anaesthesia, centrifuged, and plasma separated for validated quantitative OTA analysis contracted to the Central Science Laboratory, York, where original quality assessments were also made.

### Histology

Autopsy was performed on all animals found dead, euthanized when clinically necessary or on completion of 2 years of dietary OTA treatment. Kidneys and other tissues of potential histopathological interest were variously in whole or part either frozen in liquid nitrogen (subsequently archived at −70°C) or fixed in buffered formalin and processed automatically for 3 µm-sections and stained with haematoxylin and eosin (H & E) at the Breast Pathology Laboratory, Guy’s Hospital, London.

## Results

**Figure 1.**
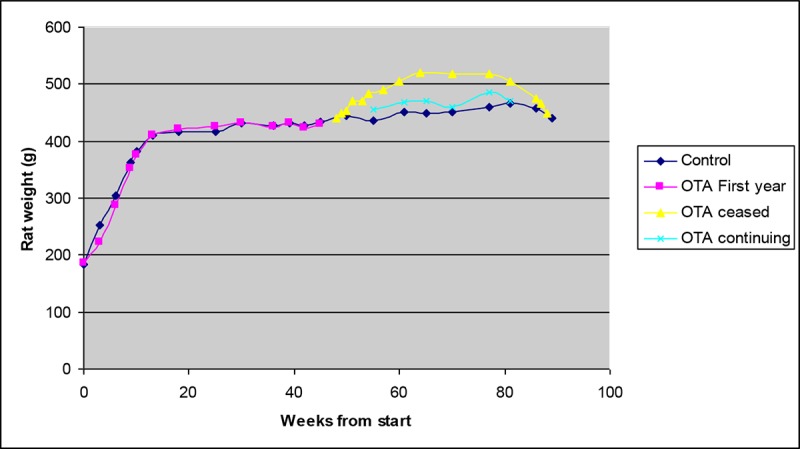
Comparative mean body weight in control and OTA-treated groups from the beginning of dietary OTA exposure (10 weeks old), showing matching of treated rats and controls during the first 10 months and significant increase to a new maximum in the sub-group that ceased OTA at that time.

**Figure 2.**
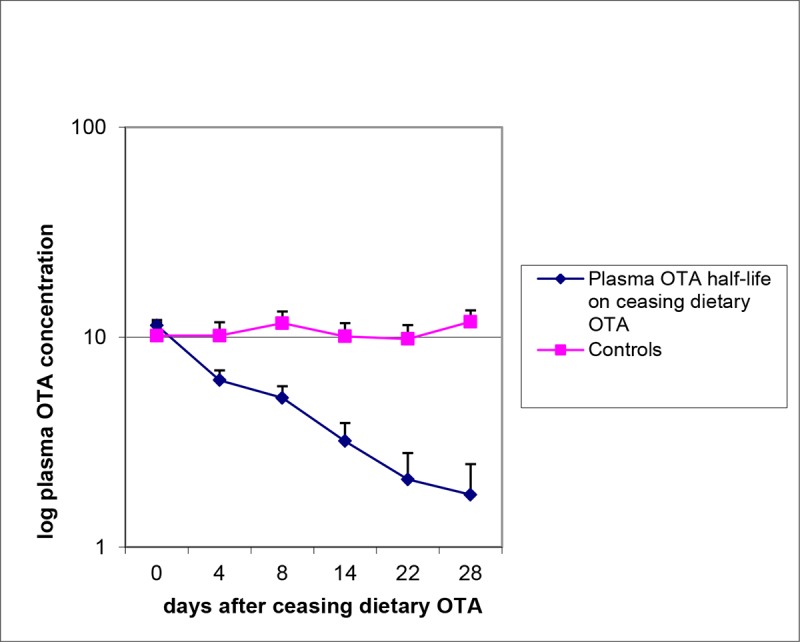
Comparative changes in plasma OTA concentration in groups of five rats during the month after either continuing or ceasing 10 months of daily exposure to 300 µg OTA/kg b.w. The slope of the ceasing group corresponds to a plasma half-life of 10 days.

Transferring young rats from standard rat diet to the OTA-contaminated experimental diet may have contributed to the initial diminution of growth rate (**Figure 1**), but this was only temporary. Otherwise, the OTA diet was always fully consumed, and growth of OTA diet-treated rats during the first year corresponded closely to that of controls. Thereafter, the weight gain in the 10-month OTA group was probably a significant response to healthier feed, while rats on continuous OTA just maintained weight. A commentary on OTA pharmacokinetics in the middle of ‘lifetime’ exposure to the contaminated diet is evident in **Figure 2**, showing steady decline in plasma OTA concentration from the steady state of ~11 µg/ml when the 10-month exposure to OTA ceased, corresponding to a plasma half-life of about 10 days.

**Figure 3.**
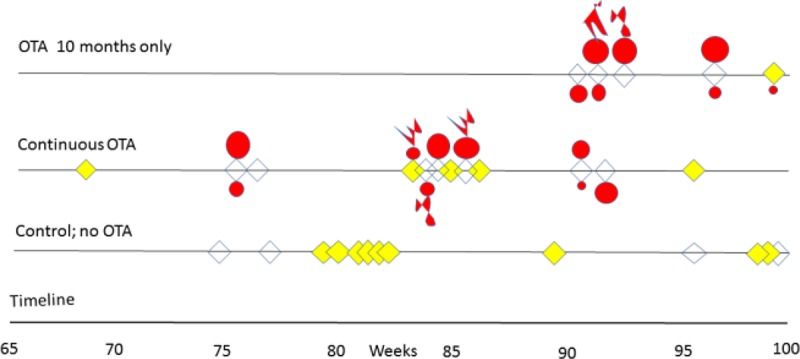
Course of outcomes for longevity, incidence of leukaemia, occurrence of renal tumours and distant metastases in male F344 rats given dietary ochratoxin A (300 µg/kg b.w.) from 8 weeks old continuously or for only 10 months, displayed from week 65 and compared with controls. Yellow indicates leukaemia, and red shows renal cancers with metastatic extension where appropriate.

Notably, OTA exposure had no adverse effect on longevity (**Figure 3**), by comparison with untreated controls. In the latter, high incidence (69%) of the mononuclear leukaemia that is characteristic in ageing F344 (Fischer) rats was evident, but simple reading of the findings in **Figure 3** suggests that continuous OTA ingestion may have somewhat delayed or prevented leukaemia (42%). Notably, inclusion of the 10-month group halves the overall leukaemia value to 35 %.

Although there was a latency of at least half-a-lifetime between cessation of the 10-month OTA exposure and discovery of renal tumours causing overt morbidity, continuous exposure appeared to be a little less carcinogenic. However, it was striking during the experiment that 75% of the continuous OTA group had already deceased before any morbidity was detected in the 10-month group. Combined incidence of kidney cancer was 70 %; there is no intention to claim any particular significance of the 100% cancer incidence in the 10-month exposure group.

**Figure 4.**
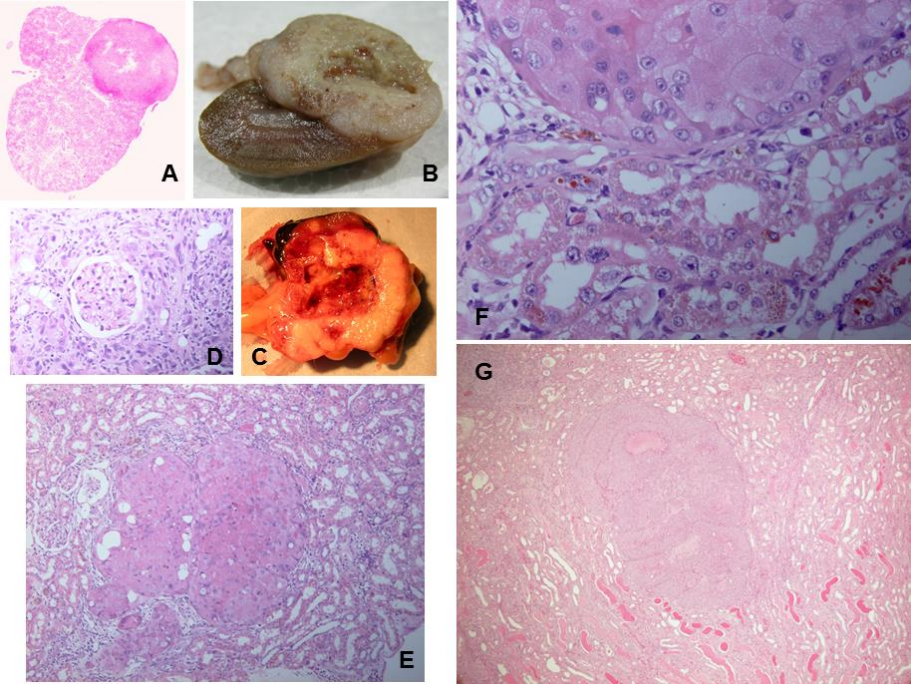
A range of rat macro- and micro-renal tumours from chronic dietary exposure to ochratoxin A. A, Longitudinal section through kidney, and tumour. B, Longitudinal half of kidney and tumour, fixed in formalin. C, Fresh half of kidney with necrotic centre and bounded by traces of original kidney. D, Glomerulus surrounded by tumour infiltrated into surrounding cortex. E, Micro-tumour located at the cortico-medullary junction. F, Junction between micro-carcinoma and kidney parenchyma showing karyocytomegaly in both tissues. G, Micro-carcinoma with small necrotic centre. B and C, Lifetime exposure to ochratoxin A; the rest relate to 10-month exposure.

**Figure 5.**
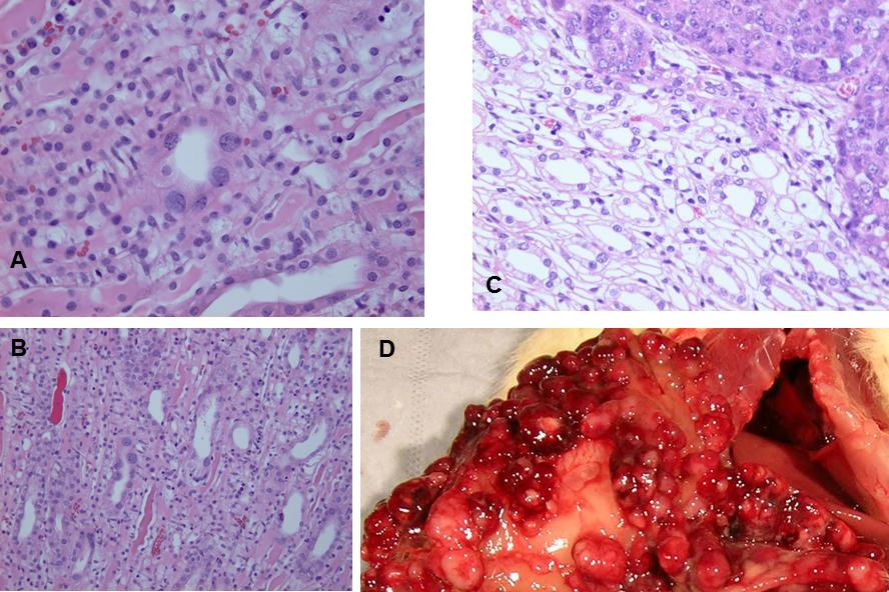
Continuous ochratoxin A. A and B, Hyperplastic renal tubule lesions in transverse and oblique sections with karyocytomegaly. C, Contrasting histology at renal tumour edge. D, Extensive nodules in serosal surfaces in upper abdomen, presumed metastatic from kidney carcinoma.

**Figure 6.**
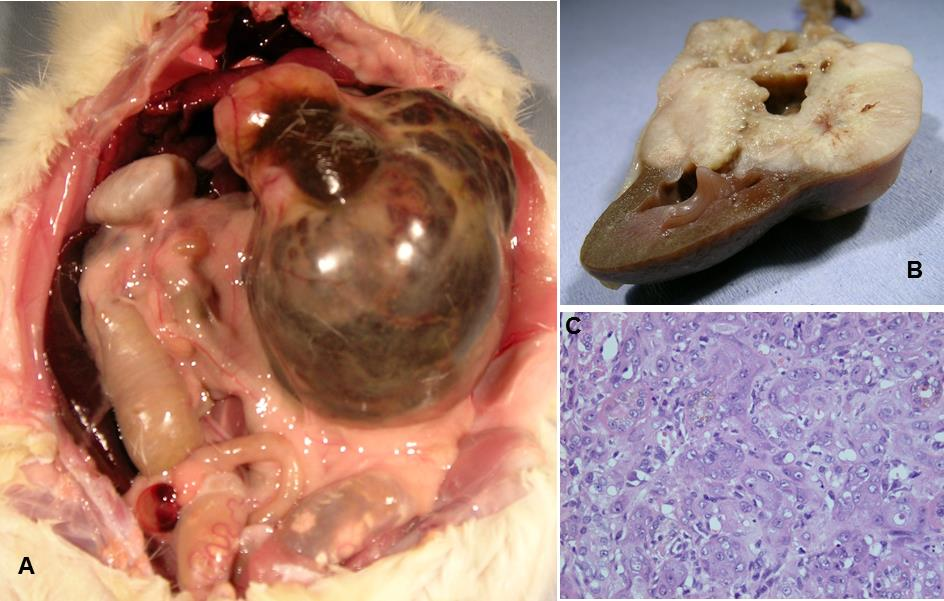
Ochratoxin A for 10 months only. A, Abdominal distortion by hydronephrotic renal cancer after 1 year of latency. B, Exposure of tumorous kidney orientated by the intact papilla and showing tumour in a discrete region. C, Detail of disorganised tumour histopathology.

No difference was perceived between the array of kidney tumours arising from 10 months of OTA intake or continued exposure for life. Conformation of kidney cancer perceived as and when animals’ well-being necessitated termination, varied widely in magnitude, uni- or bi-lateral, and the extent to which it appeared to have become a factor in clinical health. This was conditional on the propensity of Fischer rats to develop the particular mononuclear leukaemia (8). Morphology and histology are illustrated in **Figures 4–6**. Tumours apparently arose at a single focus in the region of the cortico-medullary junction. In some cases, tumour proliferation occurred by infiltration into the cortical parenchyma, destroying kidney morphology although retaining glomeruli as structural entities (**Figure 4D**); in others, the tumour retained a discrete junction with renal parenchyma (**Figure 4F**) and expanded to distort kidney morphology either within the stretched capsule or by rupturing it. Occasionally distant metastasis occurred (**Figure 5D**).

The renal tumourigenicity of OTA exposure during only 10 months eventually became apparent in all rats, but its clinical expression after about 11 months’ latency was markedly delayed relative to the continuous OTA group (**Figure 3**). This may have been, in part, due to animals being unhindered by development of leukaemia and their consequential premature demise. However, sufficient genetic change had obviously been achieved during 10 months of exposure of proximal tubule epithelia to a relentless flow of OTA molecules during toxin elimination.

Chronological timeline summaries (correlated with **Figure 3**) of gross pathology in rats given OTA are listed below. Diagnosis of leukaemia became obvious from marked splenomegaly. Microscopically, karyomegaly was evident in kidneys of all OTA-treated rats.

### For 10-month exposure

- Right kidney carcinoma at cranial pole (9.9 g). Splenomegaly.
- Left kidney carcinoma (49 g); right kidney carcinoma on contra-pelvis aspect (3 g). Metastatic nodules on abdominal serous surfaces (**Figure 5**).
- Left kidney carcinoma (11.1 g). Metastases in lungs.
- Left renal mass (~100 g) including carcinoma and hydronephrotic balloon containing ~30 ml fluid (**Figure 6**). Right kidney containing sub-capsular carcinoma (1.2 g). Splenomegaly.
- Cryptic carcinoma (2 × 3 mm) in one kidney. Splenomegaly. One testis atrophied; one hypertrophied but not tumorous.

### Continuous exposure

- Splenomegaly.
- Found dead; bilateral kidney carcinoma (left, 10.8 g; right 5.5 g).
- Found dead; extensive ascites. Kidneys morphologically normal (karyocytomegaly recognised in the outer stripe of the outer medulla). Liver very pale, possibly tumourous but not studied because of postmortem deterioration.
- Left kidney carcinoma at pole (2.4 g). Metastatic nodules on ileum surface. Splenomegaly.
- Left kidney carcinoma on contra-pelvic aspect. Metastatic nodules on abdominal serosal surfaces, in lung and in liver.
- Right kidney carcinoma (27 g).
- Splenomegaly.
- Left kidney carcinoma (8.9 g). Metastases in lung.
- Splenomegaly.
- Large abdominal sub-cutaneous sarcoma (6 × 4 cm). Left kidney carcinoma at polar aspect (4 g). Right kidney carcinoma at polar aspect (1.4 g).
- Right kidney carcinoma at cranial pole (8 g).
- Splenomegaly.

## Discussion

In comparison with findings from the previous 6-fold lower dosage experiment with a 12 % tumour incidence (6), steady-state concentration of OTA in blood plasma had risen proportionately (from 1–2 to ~11 µg/ml), corresponding both to the dose–factor difference and the subsequent 58–100% incidence of renal cancer in the two recent sub-groups (mean incidence, 70%). The 10-day plasma half-life of OTA in F344 rats means that the blood compartment ultimately controls the rate of OTA excretion through renal parenchyma. On a body weight basis the rats’ weekly dietary OTA intake in the present study was 17,500-fold greater than the current human tolerable weekly intake (TWI) value of 120 ng/kg b.w. (9), emphasising the very high and protracted experimental OTA exposure necessary to achieve their abundant renal cancer incidence. Comfortable integration of the present tumour incidence into the log dose-response relationship for OTA in the rats (2, 10) has reinforced the steepness of that curve for the Fischer strain and emphasised the nil carcinogenicity of a threshold exposure. However, natural human intake rarely approaches the prescribed TWI.

Previous use of Dark Agouti young adult males fed with dietary OTA (5-µg/g feed, delivering three times the high dose of the NTP study (1)) found no renal neoplasms after 3-month exposure; one animal with bi-lateral kidney cancer out of 20 replicates (16 %) after 6 months, and four uni-lateral kidney cancers out of 20 replicates after 9-month exposure (11). All were found at the usual natural life span of this strain. Also, the threshold value in the NTP study has been lifted to ~30 µg/kg b.w. in Dark Agouti rats (11). That strain does not have a leukaemia component to confuse diagnostic attribution of ageing mortalities, so that experimental nil carcinoma and rat-tolerable continuous exposure and weekly intake for life are still proportionately ~1750-fold greater than the human TWI value implies. The TWI value already contains a 500-fold safety factor. Therefore, whereas rat experiments are informative concerning OTA’s renal carcinogenicity, they should not be used to support undue hyperbole concerning human toxic risk from daily dietary exposures that are common only in the low ng/kg b.w. range.

Renal cancer incidence of 58% in the continuous OTA group here is twice that of a previous study (3) in which the 300-µg/kg b.w. daily dosage was held at 100 µg/rat after 4 months of the high-dose treatment. The present higher OTA exposure during the remaining 6 months of what seems a sufficient 10 months of carcinogenic exposure could account for the higher incidence of renal cancers, even if continuing exposure through a second year makes little or no difference to cancer outcomes. Although all animals of the present study together with those of the study by Mantle and Kulinskaya (6) were initially placed randomly in groups of five, no statistical significance is accorded in the present study between renal cancer incidence values in the OTA-treated groups of five (as originally designated as a group) and 12 rats (from three original groups) (**Figure 3**); there is just a general high incidence throughout.

Cumulative OTA consumption of rats in the present 10-month dosing group was ~30 mg and, in those continuing for lifetime, in the range ~50–65 mg. In an acute study (12), a single 6.25 mg gavage dose to three mature males (15 months old) caused marked temporary morbidity, but there was normal subsequent well-being and longevity and no neoplastic histopathological changes were found.

Whereas NTP historical lifetime incidence of mononuclear leukaemia in F344 rats, and that in the 1989 study controls (1), was approximately 20%, the lower incidence in females in the 1989 study at mid- and high-OTA dose (4% and 6%, respectively) is intriguing. Notably, in spite of the OTA exposure, though insensitivity to carcinogenicity for males, their longer life still failed to match the leukaemia incidence of controls. However, incomplete data collection on that topic precluded statistical analysis. In males, leukaemia was 14% across all OTA dose rates, but these values could not support any firm conclusion about a protective effect of OTA exposure. In the London study, two decades later, there was a 70% leukaemia incidence in ageing male Fischer rats, but half this value across the 17 animals given OTA (300 µg/kg b.w.) for at least a year, and an intermediate value of 50% for the rats given the 50-µg/kg b.w. regimen (6). In an analogous study (13), 24 male rats ingested ~250-µg OTA/kg b.w. for 35 weeks from 50 weeks of age (cumulative ~24 mg OTA). Plasma OTA reached 8 µg/ml within 4 weeks. Leukaemia incidence was 38%, associated with four cases of kidney adenoma but there was no cancer. It is tantalising to consider whether this evidence points, together with that in the NTP study, to a protective effect on leukaemia of chronic OTA ingestion, including at a renal tumourigenic exposure rate. Such protective magnitude is of an order much welcomed for pharmaceutical medications. It may also be contemplated why OTA exposure in the latter year (13) was so weakly tumourigenic, more closely matching the response of female rats to OTA (1). The hypothesis of augmented glomerular transport of OTA bound to a urinary globulin and absorbed into proximal tubule epithelia by male rats (14) might apply here. The alpha2u-globulin is synthesised only in the male in response to testosterone and synthesis will decline with ageing, potentially reducing OTA’s augmented transfer into nephron epithelia.

The long latency of rat renal tumourigenesis shows that OTA is not required for tumour enlargement and proliferation, but many months of exposure above a threshold daily intake of about 30 µg/kg b.w. are needed in the first year of life for the initiation of specific changes in one or a few proximal tubule segments. Human dietary exposure to OTA, usually in very small amount, is reflected in blood (including that of the author) in trace amounts by sophisticated analysis (not available for the NTP study of the 1980s) and OTA-DNA adducts can be detected in some tissues by ^32^P post-labelling methodology. However, it is fantasy to employ homeopathic-like philosophy to ascribing a human renal carcinogenic property to OTA, without support of human exposure matching necessary experimental findings. There remains no experimental evidence that OTA can cause human urothelial cancer such as that associated with the Balkan endemic nephropathy (BEN); OTA-DNA adducts may be found in such tumours but only indicate exposure, and as yet do not prove tumour initiation.

To illustrate that rat responses do not automatically translate to the human, and concerning the renal karyomegaly found in OTA-treated rats, it is difficult to ignore contrasting findings in another mycotoxin-induced nephrotoxicity. This focuses on proximal tubules in the outer stripe of the outer medulla (OSOM) in response to a *Penicillium polonicum*–moulded wheat extract, causing striking karyocytomegalic changes in the rat but no response in a vervet monkey (15). Karyomegaly is widespread within OTA renal tumours (1). These nuclei have a complex DNA ploidy distribution within a range in which all without a regular multiplicity of the genome are aneuploid and are therefore potentially unstable (16). During continuous exposure to dietary OTA, karyomegaly frequency increases over many months within parenchyma of the OSOM (3, 17), but this does not necessarily indicate that carcinoma has yet been initiated. In *P. polonicum* nephropathy, karyomegaly is accompanied by apoptosis, as revealed by ApopTag Direct analysis (18). However, OTA treatment did not cause significant apoptosis, suggesting that OTA is unlikely to be involved in the progressive bi-lateral renal atrophy of BEN. Further, recent immunohistochemical study of rat OTA tumours (19) shows that they resemble the unique spontaneous tumours of the Eker rat and may not therefore be immediately predictive of what might happen in human kidney and are even less likely to mimic the carcinomas in some BEN patients even if some of these neoplasms occur in urothelium within the renal pelvis. Hence, the recent opinion (20) that OTA should officially be reclassified as carcinogenic to humans seems ill-judged on the basis of such sparse evidence, ignoring rigorous principles of disease epidemiology (21, 22).

While focusing here on kidney cancer, the precise origin of carcinoma(s) in other organs/sites arises because the literature since the NTP report (1) has consistently assigned these to being metastatic from the kidney tumour. While the assumption may be correct, it has not been subject to immunohistochemical verification. The matter is currently a work in progress, based on the present experiments and of those lodged in the NTP Archives. Further, since full DNA exome sequence data have now been obtained from archived frozen cancers of the present rat study, interpretation is also currently a work in progress. It is important to seek matching genome change in rats and humans to address current poorly informed speculation.
